# Variable Selection for Assessing Risk Factors for Weight and Body fat Gain During the First Year After Kidney Transplantation

**DOI:** 10.1177/15269248221122891

**Published:** 2022-09-22

**Authors:** Gabriela Schmid-Mohler, Laura Huber, Thomas F. Mueller

**Affiliations:** 1 Center of Clinical Nursing Science, 27243 University Hospital Zurich, Zurich, Switzerland; 2 Division of Nephrology, 27243 University Hospital Zurich, Zurich, Switzerland

**Keywords:** evidence-based practice, body weight changes, kidney transplantation, behavior

## Abstract

**Background:** Body fat and overall weight gain are common after kidney transplantation and are associated with poor clinical outcomes. Therefore, identification of at-risk patients is relevant for preventive interventions. **Clinical Question:** What variables influence weight and fat gain in patients in the first year after kidney transplantation? **Literature Search** Prospective and retrospective cohort studies published in or after 2001 naming fat and/or overall weight gain during the first year after kidney transplantation as outcome variable(s) were systematically searched in Medline/Pubmed in November 2018 and March 2022. **Clinical Appraisal:** We identified 16 studies examining a wide variety of potential factors influencing weight and fat gain over the first posttransplant years. These included genetic, socio-demographic, behavioral, biomedical, psychological and environmental factors. For a number of variables, study results were contradictory: some studies indicated preventive impacts on weight or fat gain; others concluded that the same factors increased it. Cases were discussed with 2 clinical experts. We eventually agreed on 13 potentially relevant risk factors for post-transplant weight/fat gain: age, gender, genes, income, ethnicity, education, eating habits, physical activity, smoking cessation, baseline BMI, baseline fat, depression and perceived overall wellbeing. **Integration into Practice** Before integration into clinical practice, a critical evaluation of all potential risk factors’ suitability for assessment will be necessary. In addition to feasibility, operational definitions and measurement methods must also be considered. **Evaluation:** To reduce the list of risk factors to the most relevant, a first testing within a prospectively collected data set is planned.

## Background / Significance of the Problem

Body fat and overall weight gain are common after kidney transplantation, and are known risk factors for poor clinical outcomes such as mortality.^1^ Even in patients with normal pre-transplant weight, unmonitored weight gain can lead to overweight or obesity.^2^ Therefore, early identification of patients with a high risk for post-transplantation weight or body fat gain is important, as it allows timely initiation of preventive interventions. To identify risk factors for weight/fat gain in the first year after kidney transplantation, an evidence-based approach was chosen.

## Clinical Question

Our clinical question was “What variables influence body fat and overall weight gain during the first year after transplantation?” To ensure that causality could be inferred, only longitudinal studies were included.

## Search of the Literature

Medline / Pubmed were searched in November 2018 that included longitudinal prospective and retrospective cohort studies that investigated risk factors for weight gain, body fat gain, or body mass index (BMI) outcomes. Our initial returns were updated in March 2022 using the following search strategy:

1. MeSH DESCRIPTOR Risk Factors
2. MeSH DESCRIPTOR Kidney Transplantation
3. MeSH DESCRIPTORs Body Mass Index OR Obesity OR Overweight OR Obesity, Morbid OR Weight Reduction Programs OR Ideal Body Weight OR Weight Loss OR Weight Gain OR Body Weight Changes OR Body Weight Maintenance OR Resistance Training OR Waist-Height Ratio OR Body WeightAnorexia OR Anorexia Nervosa
4. Underweight
5. 3 OR 4
6. 1 AND 2 AND 5

The literature search yielded 533 articles, the reference lists of which led to 5 more, resulting in a total of 538 articles. Studies were excluded if any of the following criteria applied: No prospective or retrospective cohort design with body weight as outcome (N = 500); review or protocol for future study (N = 4); reporting inconsistencies (N = 1); no measurement at month twelve (N = 10); influencing variables not measured before month twelve (N = 3); not restricted to kidney transplantation (N = 1); or the operationalization of the outcome variable(s) does not allow the drawing of inferences regarding the influencing variable (N = 2); language of publication neither English nor German (N = 1).

## Clinical Appraisal

### Results of the Literature Review

Of the 538 studies returned by the search, 16 fulfilled the criteria for inclusion in the narrative review. Of those, only 1 focused on body fat gain^3^; the other 15 explored risk factors for weight gain. All included studies were published between 2001 and 2020; the majority (N = 11) were conducted in the United States. Their common strength was their prospective (N = 11) or retrospective (N = 5) cohort design, which clarified the causality both of factors and of outcomes. Their most common limitation was a small sample size: 7worked with fewer than 100 transplant recipients.

The selected studies investigated a wide variety of risk factors on weight gain over the first years posttransplant. These included genetic, socio-demographic, behavioral, biomedical, psychological and environmental variables. The study results were synthesized narratively. Based on the evidence presented, the risk factors were divided into 5 groups:

**Identified risk factors:** Genes, lower levels of physical activity, living donation and baseline BMI.
**Not identified as risk factors:** Creatinine clearance, pretransplant diabetes, dialysis modality, the presence of calcineurin inhibitors, and haemoglobin levels.
**Risk factors with controversial results, ie, variables for which some studies reported effects, while others did not or even noted contrary effects***:* age, ethnicity, income, gender, eating habits, rejection episodes, prednisone dose, immunosuppressive regimen, dyslipidemia, hypertension, food availability, depression.
**Risk factors with very weak evidence, ie, those for which only one study found an effect:** number of hospitalization episodes, perceived overall well-being, oxidative stress, and trunk fat.
**Risk factors with no evidence in kidney transplantation:** For education, smoking cessation and creatinine before month 12, no evidence exists regarding kidney graft recipients.

### Expert opinions

All factors, but especially those with controversial or weak evidence, were discussed with 2 clinical experts independently. Both experts had broad clinical and research expertise in weight gain and solid organ transplantation, held university degrees (PhD or MD) and had published in this field of research. Any variables that at least 1 expert associated with increased risk were counted as potential risk factors. Of the risk factors identified in the literature or those not previously identified as risk factors, the experts assessed only living donation differently from the published evidence: both saw a living donation as associated with better well-being; therefore, the experts recommended not to treat it as a risk factor.

The results of the literature review and expert opinion are displayed in Table 1. Age, gender, genes, income, ethnicity, education, eating habits, physical activity, smoking cessation, baseline BMI, baseline body fat, depression and well-being were identified as potentially relevant factors for posttransplant weight and/or body fat gain.

**Table 1.** Risk Factors for Increased Weight (Kg), BMI or Body Fat Percentage at 1-Year Posttransplant.

Category	Topic	Literature review	Expert opinion assessment
**Genetic factors**	Genes	Identified as risk factor^4^	Risk factor
**Socio-demographic factors**	Age	Controversy: Younger age as risk factor,^4–8^ no effect^9–11^	An important moderating factor
	Ethnicity	Controversy: Black ethnicity (vs all other ethnicities) as risk factor for weight gain,^5, 7^ no effect of black ethnicity (vs all others) on weight gain^6, 12^	Risk factor
	Income	Controversy: Lower income as a risk factor for weight gain,^7^ higher income as risk factor for weight gain^6^	Risk factor in general population
	Gender	Controversy: Female gender as risk factor for weight gain at month 12,^5, 7, 8^ no effect of gender (either female or male) on weight gain^6, 9, 12^	Risk factor in general population
	Education	No evidence in kidney transplantation	Risk factor in general population
**Behavioural factors**	Eating habit	Controversy: Self-reported carbohydrate consumption was a risk factor for weight gain in the multivariate model,^6^ other indicators such as baseline kilocalories of energy, total fat, total carbohydrates and total protein on weight gain were identified as risk factors only in simple correlation.^6^ Self-reported percentage of fat intake, percentage carbohydrate, percentage protein intake was not identified as risk factor in either simple or multivariate analyses.^6^ Self-reported intake of mono- & disaccharides, intake of energy-rich drinks/dairy, and vegetable intake were identified as risk factors for fat gain only in mean difference analysis.^3^ Eating habits are known to be the main factor for weight gain, but the applied self-report methods in those studies may not be specific enough.	Risk factor
	Physical activity	Identified as risk factor: Lower number of steps per day (assessed with pedometer) and lower self-reported physical activity (time × intensity) as risk factors for fat gain.^3^ Lower number of minutes of moderate-to-vigorous intense activity or more time spent sedentary (assessed with pedometer) were not significantly associated with fat gain. Self-reported physical activity, days of activity and time sleeping were not identified as risk factors for weight gain in a simple correlational design.^6^	Risk factors in general population
	Smoking cessation	Lack of evidence in kidney transplantation	Risk factor in general population, eg, Hu 2018^13^
**Biomedical factors**	Creatinine during first year / creatinine clearance	Not identified as risk factor: Creatinine clearance during year 1 was not identified as risk factor.^11^ Lack of evidence: Other studies investigated creatinine only at month 12.^5, 10^	No risk factor
	Rejection episodes	Controversy: Only one study reported having “no rejection episode” as a risk factor for weight gain,^7^ whereas others did not detect a significant association^5, 10, 11^	No risk factor, having no rejection may be linked to higher wellbeing
	Hospitalization episodes	Weak: Lower number of hospitalizations is a risk factor for weight gain^8^	No risk factor
	Baseline BMI (at day of transplant)	Identified as risk factor: Higher BMI^5^ or obesity (BMI > 30)^14^ as risk factor; lower BMI^8, 10^ or BMI below 25^14^ as risk factor	Risk factor
	Prednisone dose	Controversy: Cumulative dose^11, 15^ or withdrawal after 100 days^16^ were not identified as risk factors in some studies, whereas cumulative dose^10^ and steroid withdrawal after 7 days^14, 17^ were identified as risk factor in other studies.	No risk factor
	CyA/Tac	Controversy: Type of immunosuppressive medication has effect on weight gain,^10^ whereas others do not^5^	No risk factor
	Oxidative stress	Weak: Identified as risk factor in one study (only two group comparison, not regression model)^18^	No risk factor
	Dyslipidemia	Controversy: effect of total cholesterol, whereas other parameters (egHigh density lipoprotein) were not^6, 10^	No risk factor, but associated with metabolic syndrome
	Higher trunk fat	Weak: Identified as risk factor^6^ Body composition parameters are more specific than BMI, therefore, must be included.	Risk factor, ATM may be more sensitive than BMI
	Donor type	Weak: Living donation identified as risk factor^5, 8^	No risk factor, wellbeing is probably the moderating factor here.
	Pretransplant diabetes / glucose	Not identified as risk factor^5–7, 10^	No risk factor, but is associated with metabolic syndrome
	Dialysis modality	Not identified as risk factor: Peritoneal dialysis may be protective for weight gain,^11, 15^ but due to differences in baseline weight (peritoneal dialysis patients have higher baseline weight), no risk factor (implicitly)^10^	No risk factor
	Hypertension	Controversy: Hypertensive diastolic value as risk factor, whereas systolic value is not a significant risk factor^10^	No risk factor, but is associated with metabolic syndrome
	Calcineurin inhibitor	Not identified as risk factor: No effect^15^	No risk factor
	Haemoglobin	Not identified as risk factor: No effect^10^	No risk factor
**Psychological factors**	Depression	Weak: Center for Epidemiologic Studies Depression Scale (CES-D) score had no effect on weight gain^6, 19^	Risk factor in general population
	Well-being	Weak: Identified as risk factor^6^	Risk factor, may be an important moderating factor
**Environmental factors**	Food availability	Controversy: Effect of number of grocery stores within 1-mile radius on BMI change at month 12, no effect of availability of fast food restaurant or convenience stores on BMI change at month 12^9^	No risk factor, may be culture-specific

## Integration into Practice

Based on the literature review and expert opinions, a number of potential risk factors have been identified. Before these can be integrated into clinical practice, further steps are necessary. One of these is to critically appraise each identified factor's suitability for clinical use. To do that, the affordability, practicability, effectiveness and cost-effectiveness, acceptability, side-effects and safety, and equity criteria (APEASE) can provide a valuable framework.^17^ While age, gender, ethnicity, education, smoking status and BMI fulfill all APEASE criteria and may be ready for adoption into clinical practice, others do not. As a result, those gauged less suitable will be more challenging to integrate into clinical settings.

For example, while assessments of depression and well-being were administered as part of each prospective kidney recipient's assessment for registration to a graft waiting list, they may not be part of routine follow-up care. Implementing them would be resource-intensive, particularly if administered directly by a psychologist, and may not be well-received by all patients. And while the affordability, practicability, cost-effectiveness and acceptability may all be higher for a different type of operationalization and/or of measurement method, eg, self-reporting via questionnaire, those methods would also likely be less accurate. Consequently, in addition to the APEASE-criteria, each proposed assessment method's sensitivity and specificity must also be considered.

Similarly, while dual-energy x-ray (DEXA) scans are the gold standard method to measure body fat, their cost per use (including technician time) makes them unfeasible for frequent application. For routine clinical use or home monitoring, various simple, cost-effective and reasonably accurate methods of calculating body composition are available. Bioelectrical impedance analysis (BIA), for example, is very quick, inexpensive and acceptably precise, generally making it a more feasible option.^18^

## Evaluation of Evidence-Based Practice

To foster the uptake of routine assessment of risk factors for posttransplant weight and body fat gain in post-transplant care, changes in clinical practice should be tailored to the local setting. A careful analysis of that context (ie, values and beliefs, treatment guidelines, reimbursement policies) may help the interventionists engage stakeholders and decision makers from a very early stage.^19^ The assessment of body trunk fat, physical activity, depression and well-being, and eating habits requires patient participation and may even involve considerable effort on their part; therefore, their acceptance and engagement are keys to enhancing their adoption of this intervention. As implementation outcomes, patients’ and health professionals’ acceptance and adoption of the risk assessment may also function as quality indicators for the evaluation of evidence-based practice.

### Future Areas for Investigation

The aim of the literature review and expert opinion was to produce a selection of potential factors behind increases in overall weight (BMI) or body fat in the first year following kidney transplantation. It is not yet clear which of those identified have the strongest potential to predict weight gain. Therefore, the next major step will be to reduce the potential risk factors to the most relevant.

## References

[1] Chang SH McDonald SP. Post-kidney transplant weight change as marker of poor survival outcomes. Transplantation. 2008;85(10):1443-1448. doi:10.1097/TP.0b013e31816f1cd3 18497685

[2] Beckmann S Nikolic N Denhaerynck K, et al. Evolution of body weight parameters up to 3 years after solid organ transplantation: The prospective Swiss Transplant Cohort Study. Clin Transplant. 2017;31(3):1–11. 10.1111/ctr.12896 28008650

[*3] Zelle DM Kok T Dontje ML, et al. The role of diet and physical activity in post-transplant weight gain after renal transplantation. Clin Transplant. 2013;27(4):E484-E490. doi:10.1111/ctr.12149 23758229

[4] Stanfill A Hathaway D Cashion A, et al. A pilot study of demographic and dopaminergic genetic contributions to weight change in kidney transplant recipients. PloS One. 2015;10(9):e0138885. doi:10.1371/journal.pone.0138885 26406335 PMC4583246

[5] Baum CL Thielke K Westin E Kogan E Cicalese L Benedetti E. Predictors of weight gain and cardiovascular risk in a cohort of racially diverse kidney transplant recipients. Nutrition. 2002;18(2):139-146. Doi: 10.1016/s0899-9007(01)00723-7 11844645

[6] Cashion AK Hathaway DK Stanfill A, et al. Pre-transplant predictors of one yr weight gain after kidney transplantation. Clin Transplant. 2014;28(11):1271-1278. doi:10.1111/ctr.12456 25159302 PMC4576829

[*7] Clunk JM Lin CY Curtis JJ. Variables affecting weight gain in renal transplant recipients. A J Kidney Diseases. 2001;38(2):349-353. doi:10.1053/ajkd.2001.26100 11479161

[8] Forte CC Pedrollo EF Nicoletto BB, et al. Risk factors associated with weight gain after kidney transplantation: A cohort study. PloS One. 2020;15(12):e0243394. doi:10.1371/journal.pone.0243394 33370293 PMC7769456

[9] Bloodworth RF Ward KD Relyea GE Cashion AK. Food availability as a determinant of weight gain among renal transplant recipients. Res Nurs Health. 2014;37(3):253-259. doi:10.1002/nur.21599 24805885 PMC4172572

[10] Ersoy A Baran B Ersoy C Kahvecioglu S Akdag I. Calcineurin inhibitors and post-transplant weight gain. Nephrology (Carlton. 2008;13(5):433-439. doi:10.1111/j.1440-1797.2008.00916.x 18331443

[11] van den Ham ECH Kooman JP Christiaans MHL Nieman FHM van Hooff JP. Weight changes after renal transplantation: A comparison between patients on 5-mg maintenance steroid therapy and those on steroid-free immunosuppressive therapy. Transplant Int. 2003;16(5):300-306. doi:10.1007/s00147-002-0502-1 12759720

[12] Cashion AK Sanchez ZV Cowan PA Hathaway DK Lo Costello A Gaber AO. Changes in weight during the first year after kidney transplantation. Prog Transplant. 2007;17(1):40-47. 17484244 10.1177/152692480701700106

[13] Hu T Yang Z Li MD. Pharmacological effects and regulatory mechanisms of tobacco smoking effects on food intake and weight control. J Neuroimmune Pharmacol. 2018;13(4):453-466. doi:10.1007/s11481-018-9800-y 30054897

[14] Rogers CC Alloway RR Buell JF, et al. Body weight alterations under early corticosteroid withdrawal and chronic corticosteroid therapy with modern immunosuppression. Transplantation. 2005;80(1):26-33. doi:10.1097/01.tp.0000164290.17030.bc 16003229

[15] Ducloux D Kazory A Simula-Faivre D Chalopin JM. One-year post-transplant weight gain is a risk factor for graft loss. Am J Transplant. 2005;5(12):2922-2928. doi:10.1111/j.1600-6143.2005.01104.x 16303006

[16] Elster EA Leeser DB Morrissette C, et al. Obesity following kidney transplantation and steroid avoidance immunosuppression. Clin Transplant. 2008;22(3):354-359. doi:10.1111/j.1399-0012.2008.00792.x 18279417

[17] Woodle ES First MR Pirsch J, et al. A prospective, randomized, double-blind, placebo-controlled multicenter trial comparing early (7 day) corticosteroid cessation versus long-term, low-dose corticosteroid therapy. Ann Surg. 2008;248(4):564-577. doi:10.1097/SLA.0b013e318187d1da 18936569

[18] Cho YE Kim HS Lai C Stanfill A Cashion A. Oxidative stress is associated with weight gain in recipients at 12-months following kidney transplantation. Clin Biochem. 2016;49(3):237-242. doi:10.1016/j.clinbiochem.2015.11.002 26545907 PMC4744494

[19] Stanfill A Hathaway D Bloodworth R Cashion A. A prospective study of depression and weight change after kidney transplant. Prog Transplant. 2016;26(1):70-74. doi:10.1177/1526924816632118 27136252

[20] Michie S Atkins L West R. The Behaviour Change Wheel - A Guide to Designing Interventions. Silverback Publishing; 2014.

[21] Sergi G De Rui M Stubbs B Veronese N Manzato E. Measurement of lean body mass using bioelectrical impedance analysis: A consideration of the pros and cons. Aging Clin Exp Res. 2017;29(4):591-597. doi:10.1007/s40520-016-0622-6 27568020

[22] NIC Division of Cancer Control & Population Science. Implementation Science. Accessed 12th of April, 2022. https://cancercontrol.cancer.gov/is. Accessed August 10, 2022.

